# Characterization of HBV integration patterns and timing in liver cancer and HBV-infected livers

**DOI:** 10.18632/oncotarget.25308

**Published:** 2018-05-18

**Authors:** Mayuko Furuta, Hiroko Tanaka, Yuichi Shiraishi, Takuro Unida, Michio Imamura, Akihiro Fujimoto, Masahi Fujita, Aya Sasaki-Oku, Kazuhiro Maejima, Kaoru Nakano, Yoshiiku Kawakami, Koji Arihiro, Hiroshi Aikata, Masaki Ueno, Shinya Hayami, Shun-Ichi Ariizumi, Masakazu Yamamoto, Kunihito Gotoh, Hideki Ohdan, Hiroki Yamaue, Satoru Miyano, Kazuaki Chayama, Hidewaki Nakagawa

**Affiliations:** ^1^ Laboratory for Cancer Genomics, RIKEN Center for Integrative Medical Sciences, Tokyo 108-8639, Japan; ^2^ Laboratory of DNA Information Analysis, Human Genome Center, The Institute of Medical Science, The University of Tokyo, Tokyo 108-8639, Japan; ^3^ Department of Gastroenterology and Metabolism, Institute of Biomedical and Health Sciences, Hiroshima University, Hiroshima 734-8551, Japan; ^4^ Department of Anatomical Pathology, Institute of Biomedical and Health Sciences, Hiroshima University, Hiroshima 734-8551, Japan; ^5^ Second Department of Surgery, Wakayama Medical University, Wakayama 641-8510, Japan; ^6^ Department of Gastroenterological Surgery, Tokyo Women's Medical University, Tokyo 162-8666, Japan; ^7^ Department of Surgery, Osaka International Cancer Institute, Osaka 537-8511, Japan; ^8^ Department of Gastroenterological Surgery, Institute of Biomedical and Health Sciences, Hiroshima University, Hiroshima 734-8551, Japan

**Keywords:** HBV, liver cancer, sequencing, genome integration, mitochondria

## Abstract

Integration of Hepatitis B virus (HBV) into the human genome can cause genetic instability, leading to selective advantages for HBV-induced liver cancer. Despite the large number of studies for HBV integration into liver cancer, little is known about the mechanism of initial HBV integration events owing to the limitations of materials and detection methods. We conducted an HBV sequence capture, followed by ultra-deep sequencing, to screen for HBV integrations in 111 liver samples from human-hepatocyte chimeric mice with HBV infection and human clinical samples containing 42 paired samples from non-tumorous and tumorous liver tissues. The HBV infection model using chimeric mice verified the efficiency of our HBV-capture analysis and demonstrated that HBV integration could occur 23 to 49 days after HBV infection via microhomology-mediated end joining and predominantly in mitochondrial DNA. Overall HBV integration sites in clinical samples were significantly enriched in regions annotated as exhibiting open chromatin, a high level of gene expression, and early replication timing in liver cells. These data indicate that HBV integration in liver tissue was biased according to chromatin accessibility, with additional selection pressures in the gene promoters of tumor samples. Moreover, an integrative analysis using paired non-tumorous and tumorous samples and HBV-related transcriptional change revealed the involvement of *TERT* and *MLL4* in clonal selection. We also found frequent and non-tumorous liver-specific HBV integrations in *FN1* and *HBV-FN1* fusion transcript. Extensive survey of HBV integrations facilitates and improves the understanding of the timing and biology of HBV integration during infection and HBV-related hepatocarcinogenesis.

## INTRODUCTION

Hepatitis B virus (HBV) infection leads to a variety of liver diseases, such as acute or fulminant hepatitis, chronic hepatitis, cirrhosis, and eventually liver cancer. Approximately 257-million people are chronically infected with HBV, which contributes to approximately 887,000 deaths in 2015 [1]. HBV infection causes chronic inflammation of liver tissues [2, 3], and thereby increases risk of hepatocarcinogenesis as well as other chronic hepatitis related diseases, which may also be caused by Hepatitis C virus (HCV), alcohol abuse, and non-alcoholic steatohepatitis (NASH). HBV is a DNA virus that contains 3.2 kb of partially double-stranded DNA, which codes seven proteins including the structural proteins (HBsAg) and HBV X protein (HBx). HBx controls HBV transcription from covalently closed circular DNA (cccDNA) [4]. Integration of the HBV double-stranded DNA into the human genome has been observed in cancerous and non-cancerous liver tissues, and can induce genetic damage and chromosomal instability of the host genome leading to tumor progression via oncogenic activation and/or tumor suppressor inactivation [5, 6].

In the life cycle of HBV, after virion entry into hepatocyte cells, the nucleocapsid containing relaxed circular DNA (rcDNA) is released into the cytoplasm, transported to the nucleus [7], and converted into cccDNA using nuclear host proteins. The cccDNA acts as the viral template for messenger RNAs (mRNA) and pregenomic RNA (pgRNA), which, along with the viral polymerase, is encapsulated into viral capsids in the host cytoplasm [7]. Reverse transcription of pgRNA occurs within the nucleocapsid, resulting in rcDNA or double-stranded linear DNA (dslDNA) forms. These rcDNAs and dslDNAs are mainly enveloped and secreted as virions or cycle back to the nucleus to add to, or replenish, the intranuclear cccDNA pool [7]. An additional possible fate for intra-nuclear dslDNA genome is integration into the host cell genome, which is presumed to be the source of HBV integrations into the human genome [8, 9]. However, neither the timing nor mechanism of HBV integration into human hepatocytes is well understood.

To understand the contribution of HBV integration during hepatocarcinogenesis, previous studies focused on the detection of HBV integration sites in liver cancer tissues, and NGS (next-generation sequencing) technologies have enabled and accelerated the analysis of the entire human genomes to comprehensively detect HBV integration sites in liver cancers. These methods permit preferential identification of HBV DNA integration sites in the human genome, including *TERT* and *MLL4* (*KMT2B*) [10–12]. *TERT* encodes telomerase reverse transcriptase, and it is essential to maintain telomere length and associated with cell mortality, and *MLL4* (*KMT2B*) encodes histone methyltransferase, regulating chromatin structure and gene transcription, both of which are related with carcinogenesis. However, HBV integration can occur in non-cancer liver tissues, and the integration pattern in non-cancer liver tissues has been demonstrated to be more heterogeneous than that in cancers [13, 14], hence more sensitive and comprehensive analysis methodologies are required to investigate HBV integration in non-cancerous liver tissues.

In the present study, we captured HBV sequences from the DNAs of human cancer or non-cancer liver tissues, employing ultra-deep sequencing to detect human-HBV chimeric reads indicative of HBV integration. We first used liver tissues from human-hepatocyte chimeric mice with HBV infection [15] to evaluate the efficiency of the HBV-capture procedure and examine the timing and initial characteristics of HBV-integration events in HBV-infected liver tissues. We then analyzed 86 human liver tissues derived from HBV-positive patients. The results of this analysis demonstrated genome-wide HBV integrations in both cancerous and non-cancerous liver tissues and led to the speculation that the pathological mechanism of HBV integration in HBV infection and hepatocarcinogenesis. These findings indicated that HBV integration in hepatocytes could occur in early timing after HBV infection and they was biased according to chromatin accessibility of the host genome, with additional clonal selection.

## RESULTS

### HBV sequence capture and deep sequencing in HBV-infected human-hepatocyte chimeric mouse model

**Figure 1:**
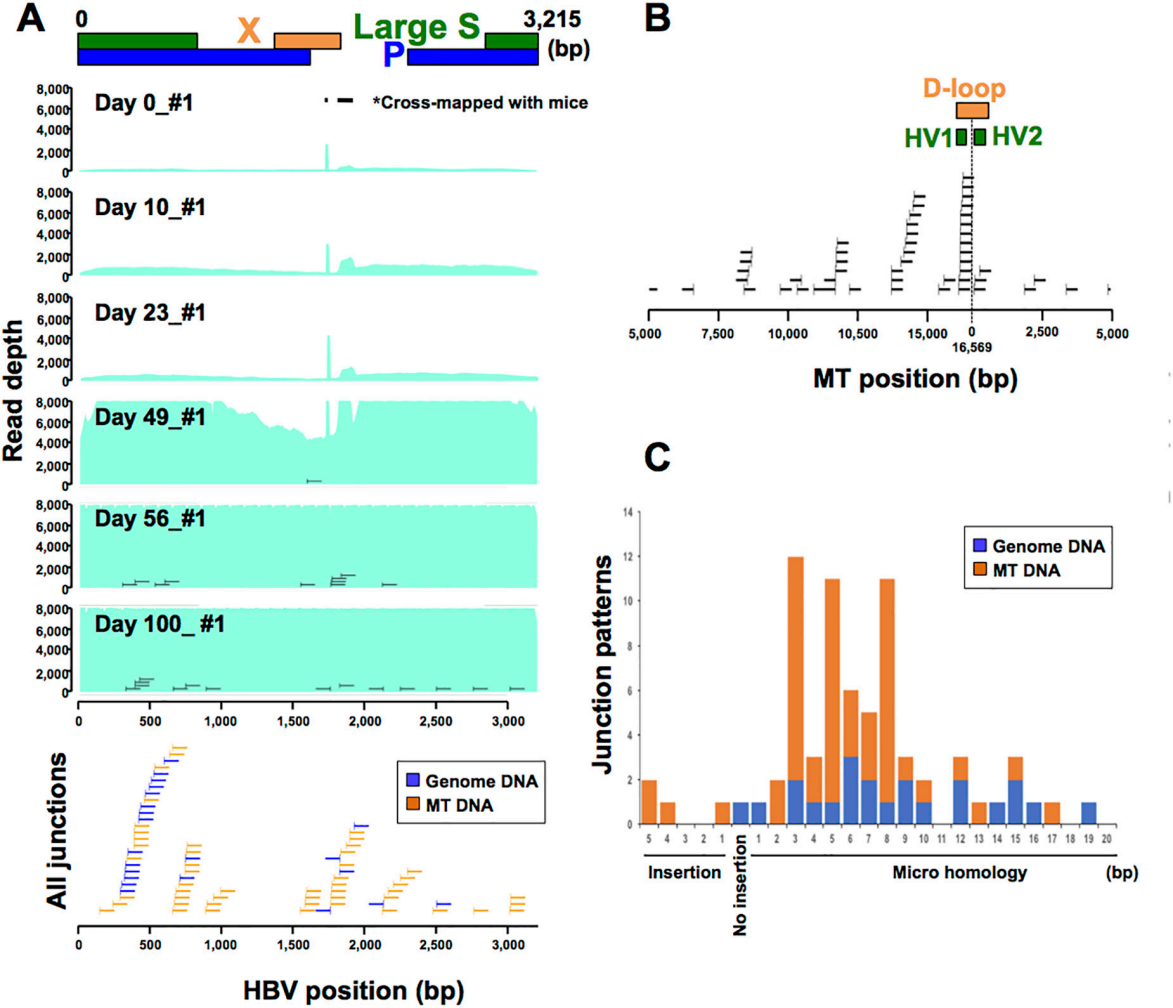
HBV infection model using human-hepatocyte chimera mice **(A)** Representative image of depth of coverage of HBV genome and integration sites in chimera mouse model 0, 10, 23, 49, 56 and 100 days after HBV infection (upper). Light green coloration indicates the HBV coverage depth. HBV junctions are indicated by vertical bars, with remaining sections of the integrated sequence indicated by horizontal lines. All junction sites and sections of integrated sequences were shown in blue (genomic DNA) and yellow (mtDNA) lines (lower). Two peaks (^*^) in HBV depth were derived from cross-mapped sequences from DNA obtained from mouse cells. **(B)** Distribution of HBV integration sites in mtDNA. **(C)** HBV junction site analysis. Patterns of HBV junction sites with insertions or microhomology in their reference sequence were shown by their length (bp). Blue and yellow indicates HBV junctions with genomic and mtDNA.

To investigate the genetic features associated with HBV integration sites in the human genome, we captured HBV sequences by custom probes, followed by ultra-deep sequencing (HBV-CapSeq). Using HBV-infected human-hepatocyte chimeric mouse model [15], we first validated the accuracy and efficiency of HBV-CapSeq and the algorithms for the detection of human-HBV junctions indicating HBV integration sites. Furthermore, we searched for the timing of HBV integration events and characteristics of HBV integration sites in the early phase after HBV infection. We extracted liver DNA from chimeric mice either in the absence of HBV infection (i.e. zero days after HBV infection) or 10, 23, 49, 56, or 100 days after HBV infection (Supplementary Table 1). DNA from non-HBV-infected livers spiked with either 10 or 100 copies of the HBV DNA (HBV-mixed sample) were also analyzed as negative controls. As expected, no human-HBV junction or chimeric read that escaped filtration were detected in the negative control HBV-mixed samples, while high amount of HBV sequences were detected in the HBV-mixed samples (Figure 1A, Supplementary Figure 1A). Interestingly, HBV integration sites (i.e. human-HBV chimeric reads) were detected after 49, 56, and 100 days of HBV infection that were not present at 0, 10, or 23 days after HBV infection (Supplementary Table 2).

### HBV integration sites in early timing after HBV infection in mouse models

HBV copy number in mouse sera started to increase between 23 and 49 days post-infection, radically increased between 49 and 56 days post-infection, and decreased between 56 to 100 days post-infection (Supplementary Table 1). Accordingly, HBV DNA sequences identified in liver tissues also dramatically increased after 49 days, as did detectable HBV integration events. These finding indicate active replication of HBV DNA and integration into liver tissues during this period (Figure 1A, Supplementary Figure 1A). Interestingly, 69.4% (50/72) of HBV integration sites were observed in human mitochondrial DNA (mtDNA) (Supplementary Table 2). Of these, randomly selected human-HBV junctions were validated by junction-specific PCR (Supplementary Figure 1B). In addition, as the sensitivity and accuracy considered of HBV-CapSeq is considered to be greater than that of junction-specific PCR, we generated a consensus sequence from overlapping DNA segments (contig sequence) from the original chimeric sequencing reads (Supplementary Figure 1C, Supplementary Table 2). Each of the contig sequences overlapped at least 24 nt of both the HBV and mtDNA sequence (median 73.7 nt) and did not map to any other genomic sequence, suggesting that these HBV-mtDNA integrations are not the result of mapping error. HBV integrations did not occurred in a specific site in HBV genome (Figure 1A). However, 32% of HBV integration sites in mtDNA (16/50) were in the displacement loop (D-loop) region, which is known as the major control site for mtDNA expression as it contains the origin of replication for the heavy DNA strand and the major promoters of transcription (Figure 1B) [16, 17]. Of note, we repeatedly detected mtDNA integration events through the same micro-homology site in independent mice, indicating the hotspot for HBV integration in mtDNA (Supplementary Figure 1C), which is probably related to replication or transcription. In addition, 65.3% of HBV integration sites (47/72) were combined through short sequences homologous between HBV and human DNA ranging from 1- to 19-bp, indicating that HBV integration was mediated through microhomology-mediated end joining (MMEJ) (Figure 1D).

### Analysis of HBV-human junction sites in clinical samples

**Figure 2:**
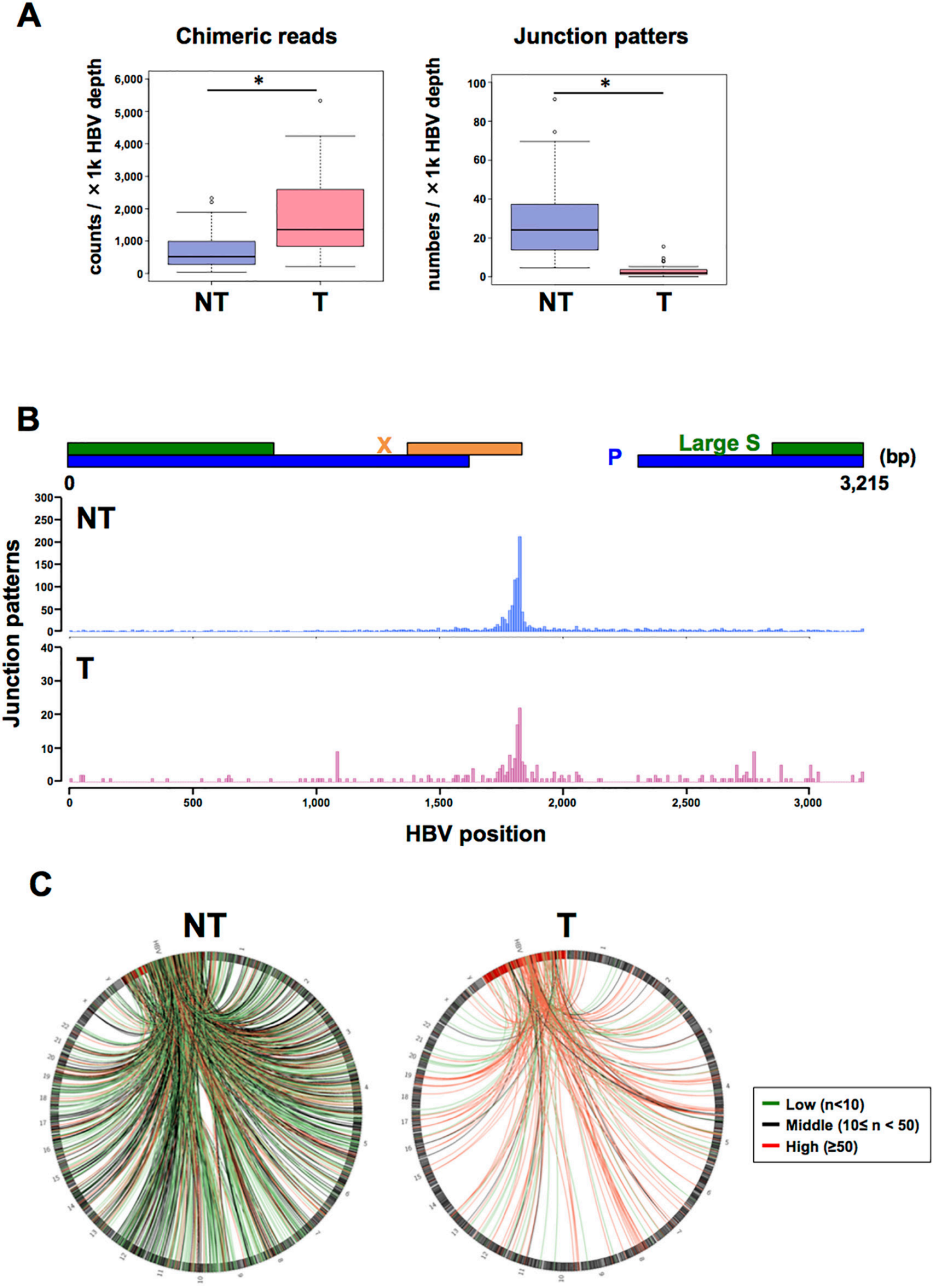
Visualization of HBV integration sites in the HBV and human genomes **(A)** The number of chimeric reads (left) and junction patterns (right) detected via HBV-CapSeq in non-tumorous (NT, blue) and tumor (T, pink) samples. ^*^p < 0.05 (paired Student's *t* test). **(B)** The number of integration breakpoints at different sites in the HBV genome. **(C)** Circos plot showing the distribution of integration sites in the HBV genome (red bars) and in human chromosomes (black bars). Red, green, and black lines indicate junctions with >50, <10, and other read counts, respectively.

Next, we used HBV-CapSeq to analyze the HBV integration sites in 42 pairs of T (tumor tissue) and adjacent NT (non-tumor liver tissue) derived from patients with potential HBV-related liver cancer. The clinical and pathological features of 40 HBV-related liver cancers and four non-HBV cases (comprising one HCV-related liver cancer, one non-HBV and non-HCV-related (NBNC) liver cancer, and two normal livers from patients with other diseases) are shown in Supplementary Table 3. All HBV-related cases showed positive for HBsAg in their sera, except for samples RK001, RK187, and RK188. HBV-DNA was detected by sensitive PCR in liver cancer tissues of each of these HBsAg-negative samples, indicating occult HBV infection. The total depth of coverage for the HBV genome was 2–7,545x in NT samples, and 2–7,874x in T samples. Finally, 4–7,033 chimeric reads, composed of 1–279 patterns of integration sites per sample, were detected in the NT samples, while 755–65,189 chimeric reads, composed of 1–34 patterns per sample, were detected in T samples (Supplementary Tables 4 and 5). The negative control samples showed no evidence of human-HBV chimeric reads after filtering. Overall, the number (normalized by total HBV sequence depth) of total chimeric reads was significantly higher, and the number of junction patterns was significantly lower, in HBV-infected T than those in NT samples. This indicates a higher clonal proportion of HBV-integrated cells in T samples than in NT samples (Figure 2A). In the HBV genome, integration sites were enriched on the 3’ side of the HBx gene, consistent with previous reports (Figure 2B) [11–14, 18]. In the human genome, integration sites were enriched on chr2, chr4, chr12, and chr19 in NT, and on chr5, chr13, chr16, and chr19 in T samples (Figure 2C, Supplementary Figure 2A-2B). We observed recurrent integrations in NT or T samples in more than four cases in 1-Mb gene regions containing genes such as *FN1, TERT, RYR2, MLL4 (KMT2B), CPS1, ANKRD17, ALB, GRIN3A*, and *LPPR1* (Table 1), some of which were consistent with previous reports [11–14, 19, 20].

**Table 1:** Frequently integrated regions (1Mb) by HBV in human genome

chr	bin_start	bin_end	case numbers	sample numbers		total junctions	genes
				liver (NT)	cancer (T)		
chr2	216,000,001	217,000,000	15	15	1	43	*FN1*
chr5	1,000,001	2,000,000	6	6	8	22	*TERT*
chr1	237,000,001	238,000,000	4	4	0	7	*RYR2*
chr19	36,000,001	37,000,000	4	4	6	17	*KMT2B(MLL4)*
chr2	211,000,001	212,000,000	4	4	0	9	*CPS1*
chr4	74,000,001	75,000,000	4	4	1	13	*ANKRD17, ALB*
chr9	104,000,001	105,000,000	4	4	0	7	*GRIN3A, LPPR1*

### Genomic features of HBV integration sites in NT and T samples

**Figure 3:**
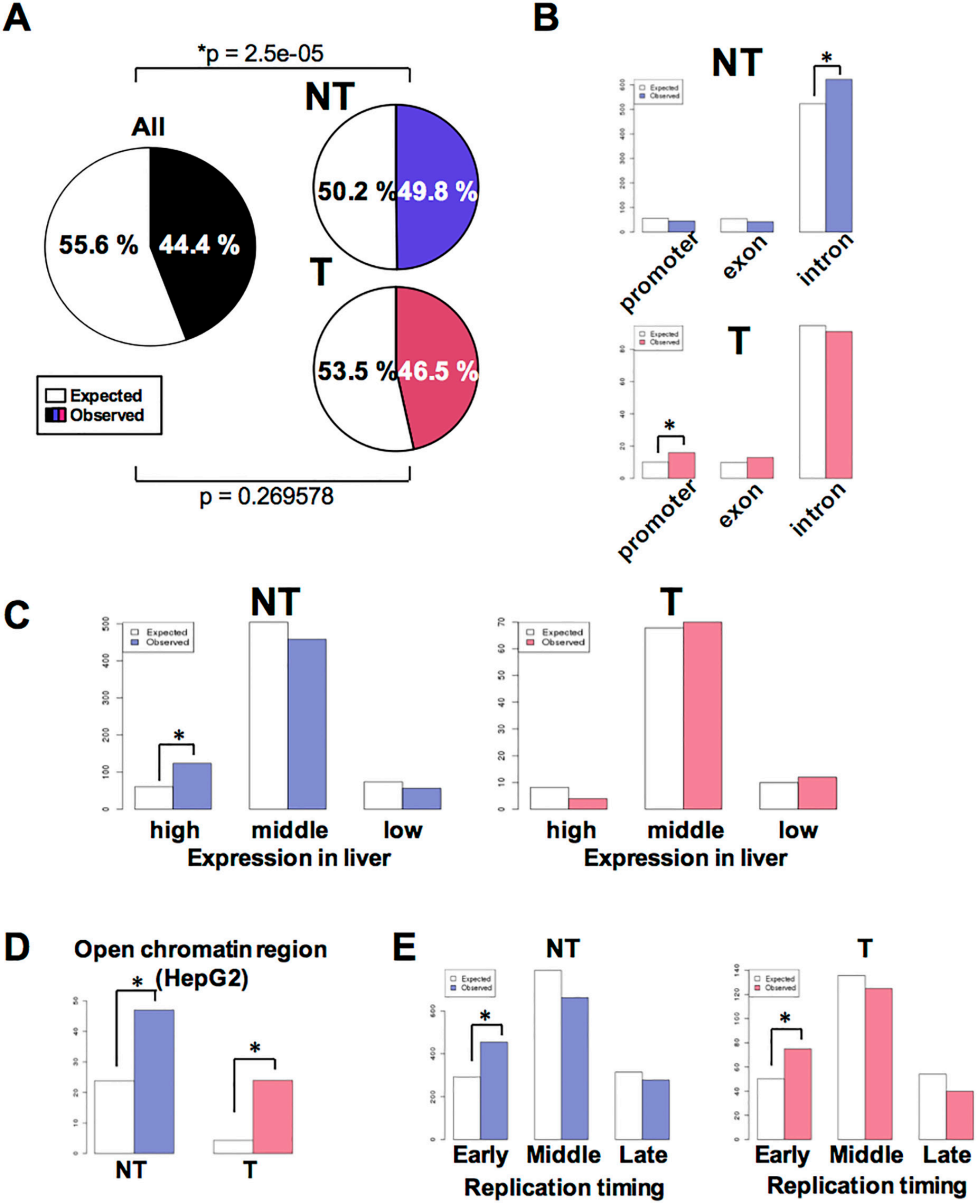
Characteristics associated with HBV integration of HBV into NT and T samples **(A)** Pie charts showing the proportion of gene body and intergenic regions in the human genome. Gene body regions were defined as exons, introns, or gene promoters (i.e., spanning 5 kb upstream to the gene transcription start site). Colored and white sections indicate the comparative proportions of gene body and intergenic junctions. **(B)** The number of HBV integration sites in gene promoters, exons, or introns among all NT and T samples. White and colored bars indicate the expected and observed number of integration sites, respectively. ^*^p < 0.05. **(C)** The number of HBV integration sites in genes found to be highly expressed in liver tissues. The expression status of the various genes in liver tissues was calculated as the mean expression level exhibited by 50 non-cancerous liver tissue samples previously described in [36]. After exclusion of genes with a Coefficient of Variation greater than 1.0, the 10% of genes that were most highly expressed and the 10% of genes that exhibited the lowest expression level were determined. ^*^p < 0.05. **(D)** The expected (white) and observed (colored) number of HBV junctions in open chromatin regions in HepG2 cell. Open chromatin regions were defined using HepG2 DNase-Seq data (https://www.encodeproject.org, GEO: GSM816662). **(E)** The expected (white) and observed (colored) numbers of HBV junctions in regions classified as ‘early,’ ‘moderate,’ or ‘late’ with regards to replication timing (defined using HepG2 Repli-seq data (GEO:GSM923446)). The ‘early’ and ‘late’ junctions were defined as regions found to exhibit greater than 70 or less than 20 gene expression signals, while those found to exhibit 20–70 gene expression signals were defined as ‘mod’.

To define the characteristics of the human genomic regions that were frequently altered by HBV integrations, we investigated the content of the integration sites with regard to the presence of functional elements. Integration sites in NT samples were significantly enriched in coding regions (particularly introns), while those of T samples were significantly enriched in promoter or upstream regions of genes. Although, integration sites were not enriched in gene body regions in T samples (Figure 3A, 3B). In NT samples, integration sites were significantly enriched in genes highly expressed in the liver, as well as open chromatin regions and regions that were classified as ‘early’ with regards to replication timing (Figure 3C-3E). In contrast, whilst integration sites in T samples showed the same pattern of enrichment of open chromatin and ‘early’ replication timing regions, they were not enriched in genes exhibiting high expression levels in the liver. Together, these results suggest that HBV integrations maintained in liver tissues are more likely to be enriched in regions encompassing genes with high expression, open chromatin, and ‘early’ replication timing regions, which explain conditions of increased chromatin accessibility. Moreover, the enrichment of integrations in T sample promoter regions suggests that there are additional clonal selection pressures exerted by HBV integrations in these specific genes.

### Comparison of HBV integration sites in paired NT and T liver samples

**Figure 4:**
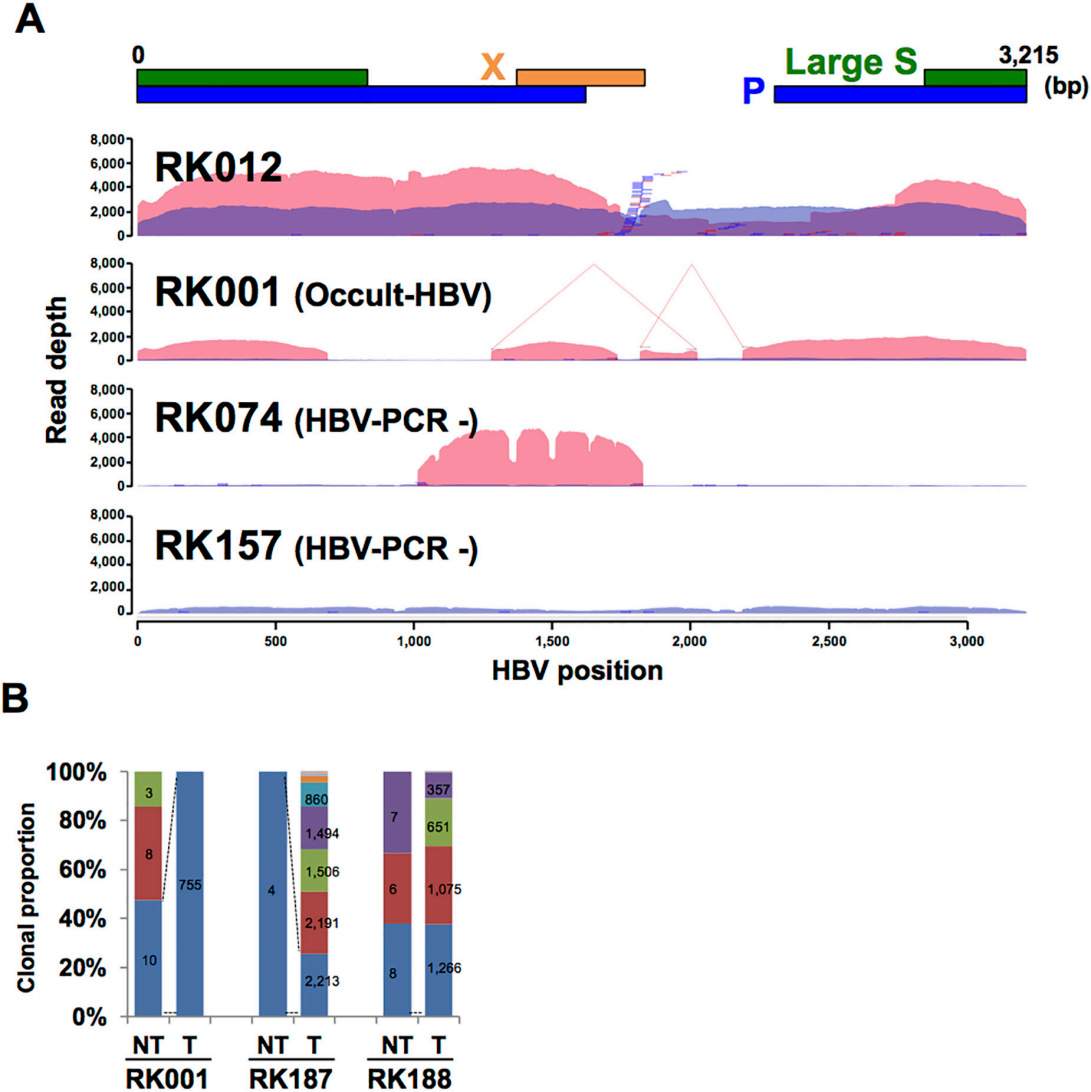
HBV integrations in paired NT and T samples **(A)** Representative image showing the depth of coverage of the HBV genome, and the integration sites identified by HBV-Capture-Seq. Blue and pink coloration indicates the HBV coverage depth in non-tumor (NT) and tumor (T) samples. Blue and red bars indicate junctions in NT and T samples. HBV junctions are indicated by vertical bars, with remaining sections of the integrated sequence (35 bp) are indicated by horizontal lines. Positions of structural variations within HBVs are depicted by connected lines. **(B)** The clonal proportion of HBV junctions in an occult HBV case. The proportion of each junction detected in the NT sample is shown, and the identically colored block indicates the shared junction between the NT and T sample. Numbers indicate the supporting read counts for each junction.

The distribution of HBV sequence depth revealed interesting patterns of HBV integration and elucidated the underlying mechanisms for observed discrepancies between HBV-CapSeq results and HBV diagnosis by other assays. For example, we observed complex HBV rearrangements in an occult HBV-infected case (RK001_T), partial integration of HBV genome in an HBV PCR-negative sample (RK074_T), and absence of HBV-integrated cells in tumors in an HBV PCR-negative sample (RK157_T) (Figure 4A, Supplementary Figure 3). Similarly, human-HBV chimeric reads were detected in both T and NT samples from three cases classified as occult HBV infection (Supplementary Table 3-4). In contrast, in four cases (RK024, RK042, RK068, and RK157), HBV integrations were detected in NT but not in T samples, suggesting that HBV integration was not a driver event for hepatocarcinogenesis in these cases. Of note, we could not find any other genetic alterations in known liver cancer driver genes (*TP53, CTNNB1, AXIN1*, or *ARID1A/2*) for these cases in our whole-genome sequencing study [11], and unknown driver events might occur to facilitate carcinogenesis in these cases.

A comparison of HBV integration sites in T *versus* NT samples revealed that the origin of clones in T samples in 37.5% (15/40) cases was independent from that of NT samples (Figure 4B, Supplementary Figure 4). Amongst the remaining cases, two cases (RK228 and RK258) were identified to harbor HBV integration sites that were shared between T and NT samples. However, the proportion of these integrations was low in T samples. The other 57.5% (23/40) were found to exhibit shared HBV-integration sites between T and NT samples, some of which were expanded in T samples, suggesting that HBV-integrated cells containing these integration sites may have been progenitors for the T samples. In all three occult-HBV cases, some HBV integration sites that were detected to have lower read counts in NT samples were shown to be expanded in corresponding T samples (Figure 4B), while four of the five HBV PCR-negative cases did not share any HBV integration sites between NT and T samples. These results indicate that rare HBV-infected NT cells in the occult-HBV cases are progenitors for the T samples. We extracted all junctions shared between NT and T samples and annotated these junctions based on the protein-coding genes (Supplementary Table 6). Among the regions surrounding 24 genes *TERT* and *MLL4* were commonly detected in T samples, indicating their importance in clonal expansion during carcinogenesis.

### Recurrently altered genes and fusion transcripts by HBV integration

**Figure 5:**
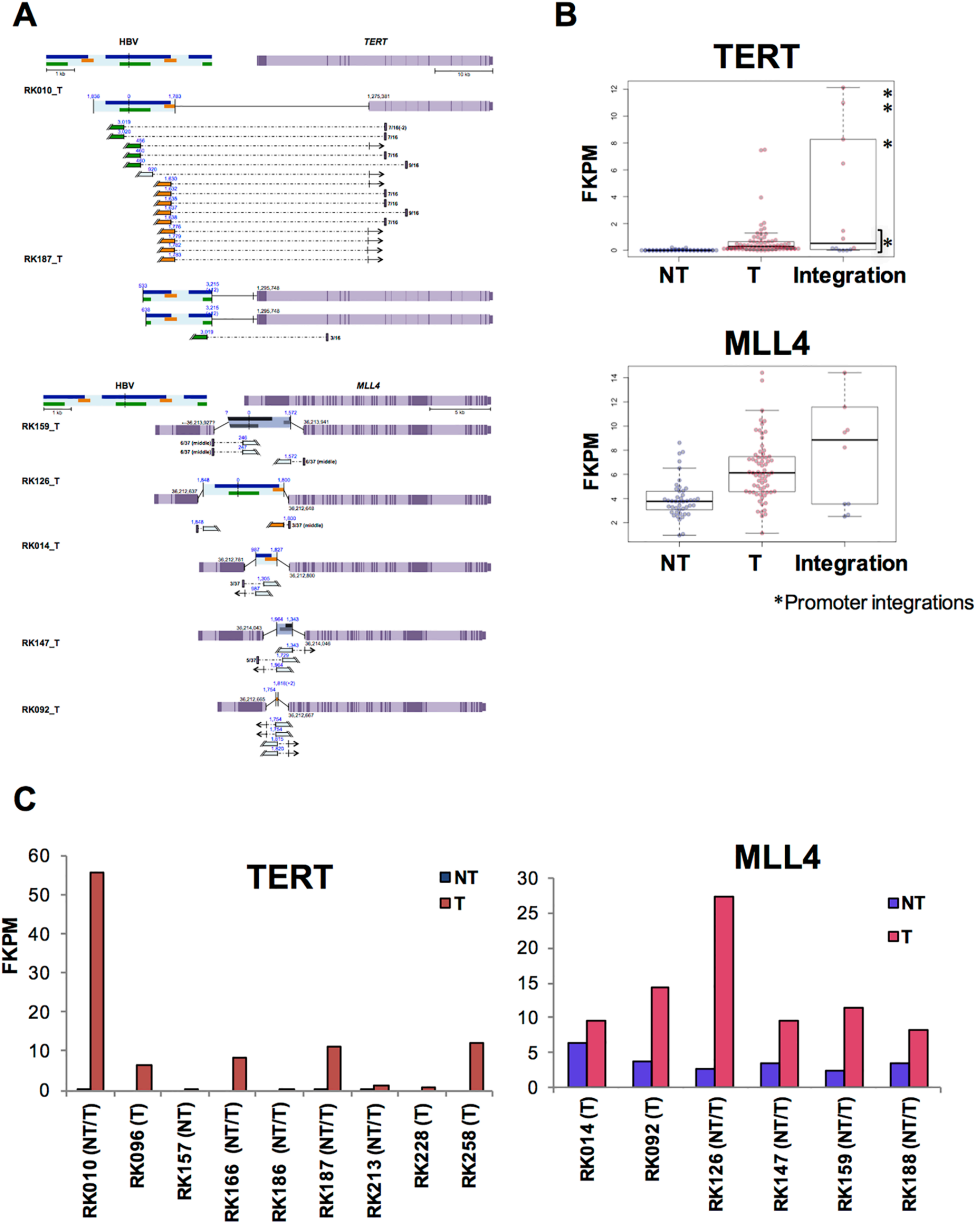
Influence of HBV integrations on *TERT* and *MLL4* expression in T samples **(A)** Expected gene structure of HBV integrations and corresponding HBV fusion transcripts for *TERT* (upper) and *MLL4* (lower). Corresponding HBV and human genomic structures are shown, including HBV genes encoding Large S (green), P (blue), and X (yellow) proteins, as well as gene exons (purple) and introns (light purple). Fusion transcripts that were determined to contain junctions via RNA-Seq are depicted (connected boxes), and the direction of gene expression (arrows) is indicated. **(B)** The expression status of *TERT* (upper) and *MLL4* (lower), as determined by RNA-Seq. Samples include both HBV integrations and, in the control, non-integrated NT and T samples. Blue and pink plots indicate NT and T samples, respectively.^*^samples with integrations in promoter regions. **(C)** Expression status of *TERT* (left) and *MLL4* (right) in integrated samples. Blue and pink bars indicate NT and T samples.

**Figure 6:**
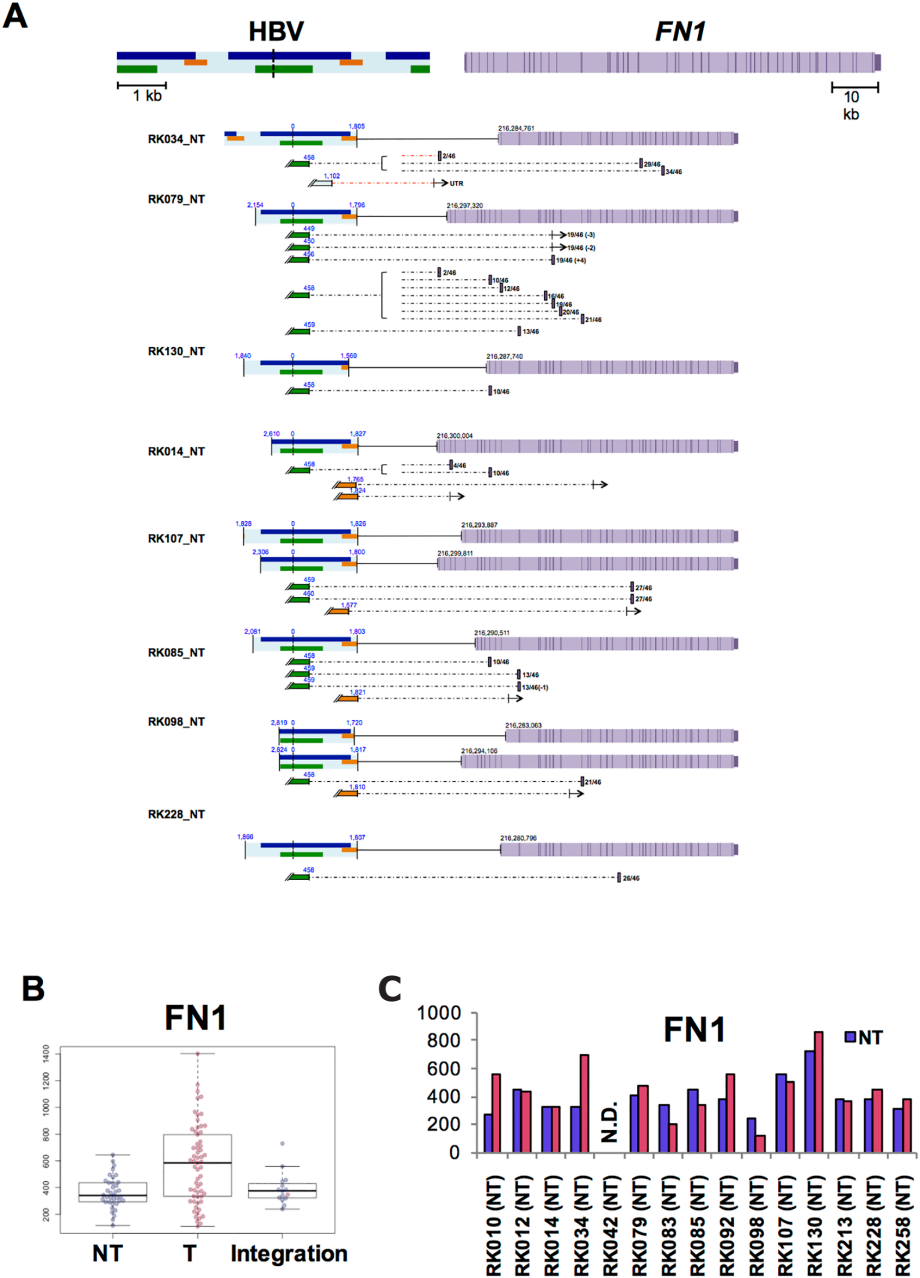
Influence of HBV integrations on FN1 in NT samples **(A)** Expected *FN1* gene structure of HBV integrations and HBV fusion transcripts. HBV genes encoding Large S (green), P (blue), and X (yellow) proteins, gene exons (purple) and introns (light purple). Fusion transcripts that were determined to contain junctions via RNA-Seq are depicted (connected boxes), and the direction of gene expression (arrows) is indicated. **(B)** Theexpression status of *FN1*, as determined by RNA-Seq. Samples include both HBV integrations and, in the control, non-integrated NT and T samples. Blue and pink plots indicate NT and T samples, respectively. **(C)** Expression status of *FN1* in integrated samples. Blue and pink bars indicate NT and T samples.

To determine the functional consequence of HBV integrations, we next investigated the effect of HBV integration on gene expression and/or transcriptional change in samples with available RNA-Seq data. Amongst the genes affected by HBV integrations, HBV fusion transcripts were detected for 27 genes, and fusions of *FN1, MLL4, TERT*, and *ALB* were detected in more than two cases (Supplementary Table 7). In addition, the expression status of *MLL4* and *TERT* in T samples with HBV integrations varied significantly (Figure 5). For *TERT*, six of eight identified HBV integrations occurred in promoter regions, and showed significantly high expression compared with other NT and T samples. One of two HBV-integration cases found to have junctions in an intron showed expression of a fusion transcript (Figure 5A-5C). Notably, promoter integration in RK187_T induced expression of an HBV-*TERT* fusion transcript. Similarly, five of the six cases observed to have junctions in either an exon or an intron of *MLL4* were induced to express fusion transcripts [21]. Furthermore, four of these five cases expressed both 5’-*MLL4*-HBV-3’ and 5’-HBV-*MLL4*-3’ fusions, suggesting that the *MLL4* mRNA sequence contains an HBV insertion, which induces overexpression of *MLL4* (Figure 5A-5C). Notably, some *TERT* and *MLL4* integration sites were detected in corresponding NT samples, albeit with low read numbers (Supplementary Figure 5), suggesting that the NT samples may contain clones harboring these integrations. In NT samples, HBV integration into *FN1* was detected in 15 of 41 cases, and eight of 14 cases expressed HBV-*FN1* fusion transcripts (Figure 6A, Supplementary Table 6). All integrations occurred in introns, and their resultant expression status was not significantly different compared to that of control NT samples. Nevertheless, detected fusion transcripts containing the HBV sequence were associated with the 5’ side of *FN1* exons, and are thus predicted to cause alternative splicing as previously reported [21] (Figure 6B).

## DISCUSSION

In the present study, we demonstrated that HBV integration occurred predominantly in human mtDNA (69.4%, 50/72) during the early phase of HBV infection after a cycle of active HBV virion replication (4-7 weeks). HBV integration does not occur immediately after initial infection but is accompanied by HBV virion secretion and at least one HBV replication cycle. These data are compatible with the hypothesis that dslDNA, which is an alternative product from viral reverse transcription of the pgRNA, could be the source of HBV DNA that is integrated into the human genome. This is the first report suggesting that HBV integrations occur in mtDNA during the acute phase of HBV infection *in vivo*. But amongst the total integration sites of human clinical liver tissues, HBV integrations in mtDNA only occurred at a rate of 0.1% (2/1,684) (Supplementary Table 5). This discrepancy could be explained by two facts: i) the immune-deficiency inherent in a mouse model, and ii) clinical samples underwent long passage and selection pressure after HBV infection. HBV integration in mtDNA could be a predominant event in the early phase after HBV infection, but they may be eliminated because of immune clearance and/or negative selective pressure. There is some evidence of mitochondrial involvement in HBV infection. Unchwaniwala *et al.* [22] visualized core (*Cp*), polymerase (*Pol*), and pgRNA during HBV infection, and identified *Pol* as localized in mitochondria. This suggests mitochondrial involvement, at least in protein stage of the HBV life cycle [22]. HBx protein is known to cause mitochondrial aggregation at the periphery of nucleus resulting in DNA damage induction and ROS generation in mitochondria via loss of membrane potential [23, 24]. D-loop mutations in the mtDNA were detected frequently in HBV-infected NT and T liver tissues [25], additionally HBV integration in the D-loop of mtDNA was also frequently detected. We found that HBV integrations were mediated through MMEJ [26, 27], which is known as an alternative DNA double-strand break (DSB) repair system and reported as a principal mediator during mitochondrial DNA lesions [28]. This suggests that early-phase HBV integration occurred in mtDNA. Damage in mtDNA is known to induce autophagy and was removed in *C. elegans* [29], therefore it is possible that integration of HBV DNA into mtDNA might be a defensive mechanism to protect the nucleus from HBV integration.

In this study, HBV integrations in NT and T clinical samples were significantly enriched in regions associated with highly expressed genes, open chromatin, and early replication timing in hepatocytes. The enrichment of HBV integration sites in the promoter regions of T samples indicates the existence of positive selective pressure during the clonal development of tumors [12, 13]. In addition, differing from our clinical samples (Figure 2B) and previous independent studies [11–14, 20], integrations did not predominantly occur in the HBx region during the early HBV infection model (Figure 1A), suggesting that accumulation of integration sites in the HBx gene was generated under positive or negative selective pressure of cells with HBV integrations.

It is important to understand the consequence of HBV integration to cancer development, therefore, comparing NT and T samples from the same patient using comprehensive analysis of DNA and RNA from same sample is important for the identification of possible candidates involved. HBV integrations were maintained and expanded from NT to T samples, and HBV integrations result in transcribed fusion transcript considered to be important for tumor development. Overall, *MLL4* and *TERT* matched both criteria and are also known as frequently altered genes in HCC [10–14, 19–21, 27], suggesting their probable involvement in HBV-infected HCCs. HBV integrations in *TERT* occurred close to the promoter, whereas *MLL4* integrations were in the gene coding sequence. HBV integrations induced a significant increase in the level of *MLL4* expression, although the mechanism underlying this increase is not clear. Interestingly, *FN1* fusion transcripts were observed specifically in NT samples. For *FN1* integrations in NT samples, the junctions of the *HBV-FN1* fusion transcripts varied but were enriched at position +458 of the HBV genome, whereas the junctions of *FN1* occurred at the exon transcription start sites. These results indicate that splicing of the HBV-*FN1* fusion transcript occurred regardless of the integration site. Interestingly, although the exons of the fusion sites varied between samples, HBV-FN1 transcripts were expected to form in-frame *HBV-FN1* transcripts. Given that *FN1* is a key modulator of fibrosis [30], the HBV-FN1 fusion protein may be involved in the pathogenesis of liver fibrosis. Furthermore. *FN1*-driver gene fusions were reported in other rare tumors such as *FN1-ALK* [31] and *FN1-FGFR1* [32] and *FN1* expression might be related with driver event for cancer, but further study is required to evaluate the functional significance of such HBV-*FN1* fusions.

## MATERIALS AND METHODS

### Clinical samples

Samples were collected from all subjects during partial hepatectomy, which also included the collection of adjacent non-cancerous liver tissues. After collection, all samples were stored at -80°C. High molecular-weight genomic DNA was extracted from these fresh-frozen tumor specimens (tumors, T) and adjacent non-cancerous liver tissues (non-tumors, NT). All subjects provided informed consent to participate in the study, which was conducted in strict accordance with International Cancer Genome Consortium (ICGC) guidelines [33], and approved by ethical committees at RIKEN, Japan, and by all participant groups. For the detection of HBV by PCR, tumor DNA was used as a template for PCR-amplification using primers targeting the HBV sequence (spanning 2,851–690 in the HBV genome; 5’-GGTCACCATATTCTTGGGAA-3’ and 5’-AATGGCACTAGTAACCTGAG-3’). PCR was performed with 2.5 ng of input DNA using ExTaq (TaKaRa, Shiga, Japan) with 35 cycles.

### HBV-CapSeq

Extracted T and NT DNA was sheared into approximately 500-bp fragments using a Covaris Ultrasonicator (Covaris, MA, USA). Preparation of libraries and target capture for HBV sequences were performed, using the SureSelectXT Target Enrichment System for Illumina Multiplexed Sequencing (version 1.5), according to the manufacturer's protocol but with some modifications to pre-capture PCR conditions. Specifically, pre-incubation cycling comprised of 98°C for 3 min, 5 cycles of 98°C for 80 s, 65°C for 30 s, and 72°C for 1 min. Hybridization comprised incubation of 750 ng of the libraries at 72°C for 10 min with the target probes and was amplified by PCR for 13 cycles. Target probes were designed from 73 HBV reference genomes [34]. The captured libraries were sequenced using the HiSeq2000/2500 platform with paired-end reads of 100-bp, 125-bp, or 150-bp, according to the manufacturer's instructions (Illumina, CA, USA). For comparison analysis, all sequencing reads were trimmed to the first 100-bp to be comparable between samples, and the original lengths were used for verification analysis.

### Detection of HBV integration

Firstly, we aligned short reads to the GRCh37 human reference sequence and an HBV sequence (Genbank Accession: AP011098) using BWAmem software (https://sourceforge.net/projects/bio-bwa/, –T 0 options). We detected HBV-human chimeric reads as previously described [35]. Briefly, chimeric read pairs comprising HBV and human sequences, including paired reads (i.e. those occurring close to breakpoints and reading in a consistent direction) and discordant read pairs (i.e. those in which one read aligns to the HBV sequence while the other aligns to the human genome), were assessed using Genomon-SV software (https://github.com/Genomon-Project/GenomonSV). Integration candidates were selected to have a number of reads > 3, and an overhang size > 100.

### Generation of human-hepatocyte chimeric mice with HBV infection

Generation of the uPA^+/+^/SCID^+/+^ mice, and transplantation of human hepatocytes, were each performed as previously described [15]. Mice underwent hepatocyte transplantation, whereby 90% of their liver tissue was replaced with human hepatocytes. Eight weeks later, the chimeric mice were intravenously injected with 5 × 10^5^ copies of the HBV virus (carried in HBV-positive human serum), and ethically sacrificed either ten days, 23, 49, 56, or 100 days after HBV infection. Their livers were extracted and their concentration of human serum albumin (HSA, used as an indicator of liver repopulation [15]), was measured as previously described [36]. All animal experiments were performed in strict accordance with both the Guide for the Care and Use of Laboratory Animals, and the guidelines stipulated by the local Committee for Animal Experiments. The experimental protocol was approved by the Ethics Review Committee for Animal Experimentation of the Graduate School of Biomedical Sciences, Hiroshima University (protocol number 12-93). Human serum samples containing high titers of HBsAg-positive genotype C HBV DNA (7.6 × 10^6^ copies/mL), were obtained from a patient with chronic hepatitis, divided into aliquots, and stored in liquid nitrogen until use. The patient provided written informed consent for the use of the collected DNA in accordance with the Declaration of Helsinki, and with approval from the Ethical Committee of Hiroshima University (approval ID: D08-9). HBV DNA levels in mouse sera were measured quantitatively using a TaqMan-based assay (COBAS AmpliPre/COBAS TaqMan HBV TEST, v2.0, Roche Diagnostics, Tokyo, Japan) according to the manufacturer's instructions. The lower detection limit of real-time PCR for HBV DNA was 4.4 log copies/mL [37].

### Datasets

To identify genes that were highly expressed in liver tissues, we used fragments per kilobase of exon per Million mapped fragment (FPKM) values from a previously published RNA-Seq dataset that was generated using 50 NT from patients with hepatocellular carcinoma (HCC) [38]. The expression status of various genes in liver tissues was calculated to be the mean expression level identified among the analyzed samples. After removing genes with a coefficient of variation greater than 1.0, we identified the 10% of genes that exhibited the highest, and the 10% of genes that exhibited the lowest level of expression in the liver tissue samples. We defined open chromatin regions and replication timing in livers using DNase-Seq data (GEO: GSM816662) and Repli-Seq data (GEO: GSM923446) for HepG2 cell lines, as listed within the ENCODE database (https://www.encodeproject.org). Open chromatin regions were identified as regions from which gene expression signals were detected, while regions defined as displaying “early” and “late” replication timing were classified as those exhibiting greater than 70 and less than 20 gene expression signals, respectively. Regions exhibiting 20-70 gene expression signals were classified as displaying “moderate” replication timing.

## SUPPLEMENTARY MATERIALS FIGURES AND TABLES




