# Correction: Characterization of HBV integration patterns and timing in liver cancer and HBV-infected livers

**DOI:** 10.18632/oncotarget.25960

**Published:** 2018-08-03

**Authors:** Mayuko Furuta, Hiroko Tanaka, Yuichi Shiraishi, Takuro Uchida, Michio Imamura, Akihiro Fujimoto, Masahi Fujita, Aya Sasaki-Oku, Kazuhiro Maejima, Kaoru Nakano, Yoshiiku Kawakami, Koji Arihiro, Hiroshi Aikata, Masaki Ueno, Shinya Hayami, Shun-Ichi Ariizumi, Masakazu Yamamoto, Kunihito Gotoh, Hideki Ohdan, Hiroki Yamaue, Satoru Miyano, Kazuaki Chayama, Hidewaki Nakagawa

**Affiliations:** ^1^ Laboratory for Cancer Genomics, RIKEN Center for Integrative Medical Sciences, Tokyo 108-8639, Japan; ^2^ Laboratory of DNA Information Analysis, Human Genome Center, The Institute of Medical Science, The University of Tokyo, Tokyo 108-8639, Japan; ^3^ Department of Gastroenterology and Metabolism, Institute of Biomedical and Health Sciences, Hiroshima University, Hiroshima 734-8551, Japan; ^4^ Department of Anatomical Pathology, Institute of Biomedical and Health Sciences, Hiroshima University, Hiroshima 734- 8551, Japan; ^5^ Second Department of Surgery, Wakayama Medical University, Wakayama 641-8510, Japan; ^6^ Department of Gastroenterological Surgery, Tokyo Women’s Medical University, Tokyo 162-8666, Japan; ^7^ Department of Surgery, Osaka International Cancer Institute, Osaka 537-8511, Japan; ^8^ Department of Gastroenterological Surgery, Institute of Biomedical and Health Sciences, Hiroshima University, Hiroshima 734-8551, Japan

**This article has been corrected:** The correct author name is given below:

**Takuro Uchida**

Original article: Oncotarget. 2018; 9:25075-25088. https://doi.org/10.18632/oncotarget.25308

