# Supplementary material for: Characterization of HBV integration patterns and timing in liver cancer and HBV-infected livers
Source: Oncotarget. 2018 May 18;9(38):25075–88. doi: 10.18632/oncotarget.25308 (PMC5982772; doi:10.18632/oncotarget.25308)
Supplement: Supplementary file 1 [file oncotarget-09-25075-s001.pdf]

Characterization of HBV integration patterns and timing in liver cancer and HBV-infected livers

SUPPLEMENTARY MATERIALS

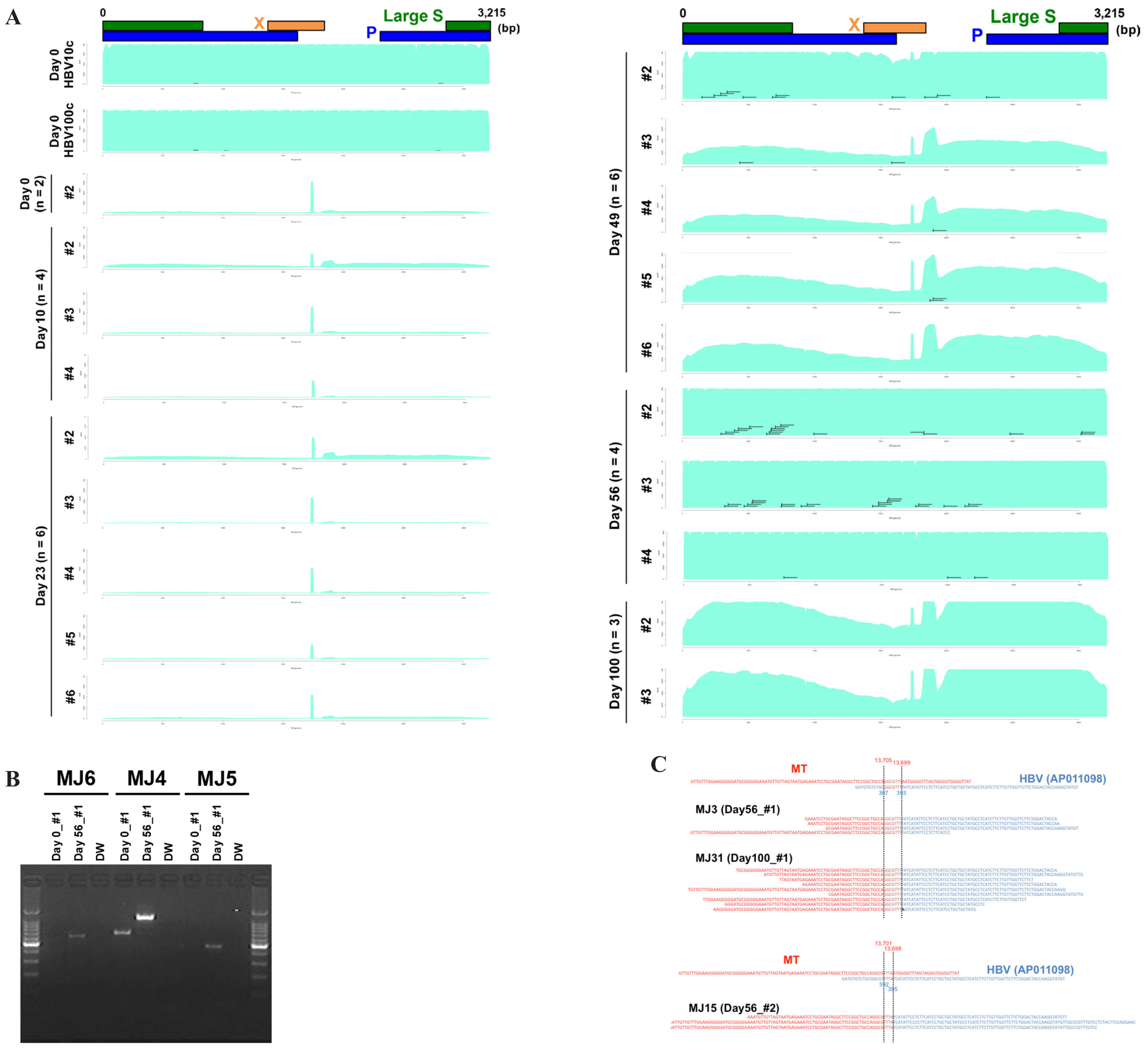

Supplementary Figure 1: (A) Coverage of HBV genome (light green) and integration sites (black bars) in HBV-Capture-Seq. Junctions of HBV are shown as vertical bars, with part of the remaining integrated sequence (35bp) indicated as horizontal lines. Remaining samples were shown in Figure 1A. **(B)** Representative example of verification of HBV integration by junction-specific PCR. Junction-specific PCR were performed as shown previously (ref). Detailed information of each junction was shown in Supplementary Table 3. **(C)** Example of break points between HBV and MT (mtDNA). Red, blue and yellow characters' indicating sequence of MT, HBV, and micro homology shared between MT and HBV. Day 56_#1 and Day 100_#1 shared identical junction sequence (MJ3 and MJ31) although there are derived from different mice, and junction MJ15 from Day 56_#2 were derived from very close combination of locus with MJ3 and MJ31.

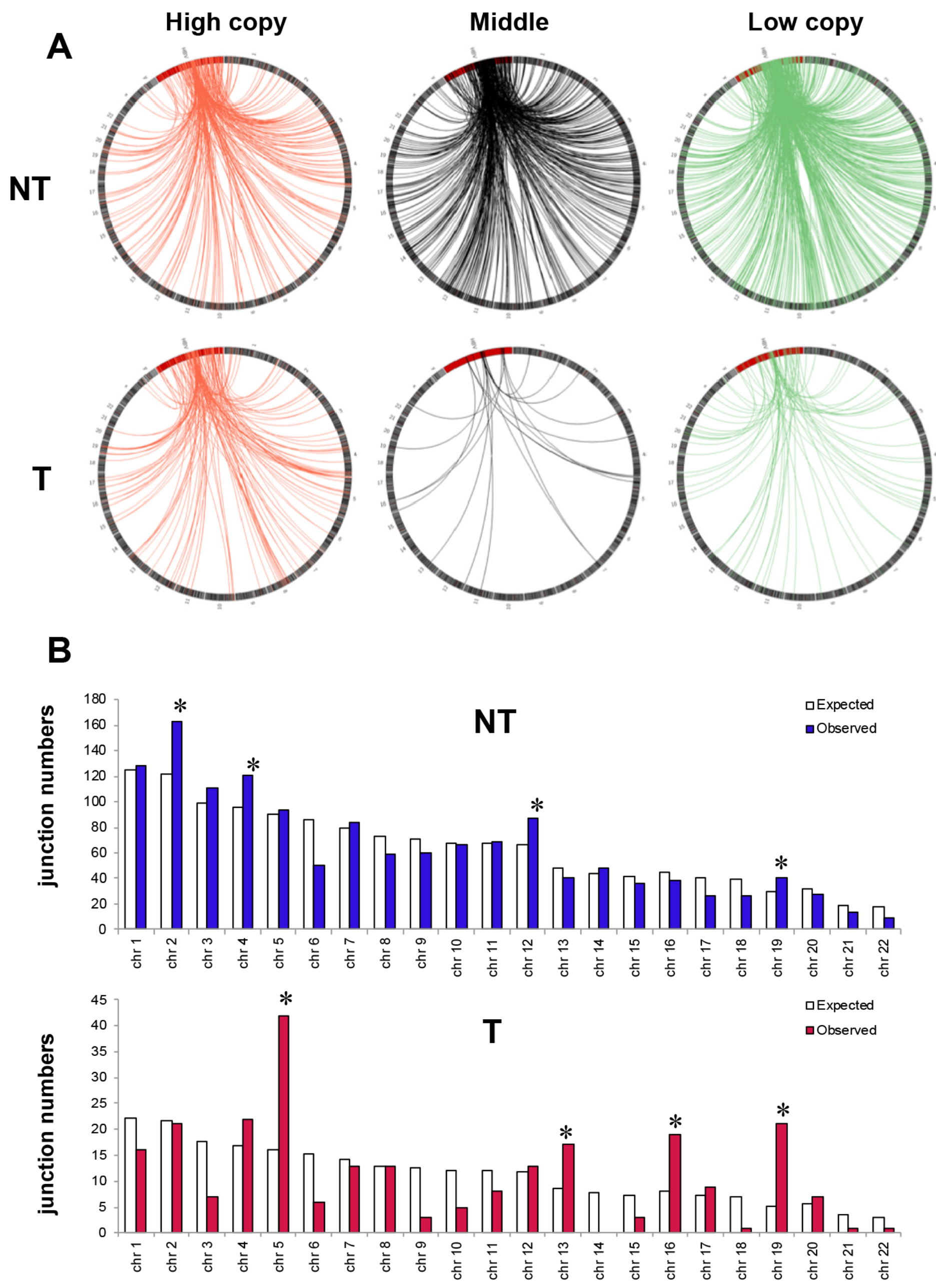

Supplementary Figure 2: (A) Circos plot showing the distribution of integration sites in the HBV genome (Red bars) and human chromosomes (Black bars). High copy, Low copy and middle indicate junctions with read counts over 50, under 10, and others respectively. **(B)** Expected (white bars) and observed (colored bars) numbers of HBV junctions in each chromosomes. Expected number of junctions were normalized with the length of each chromosome. $^{*}P < 0.05$.

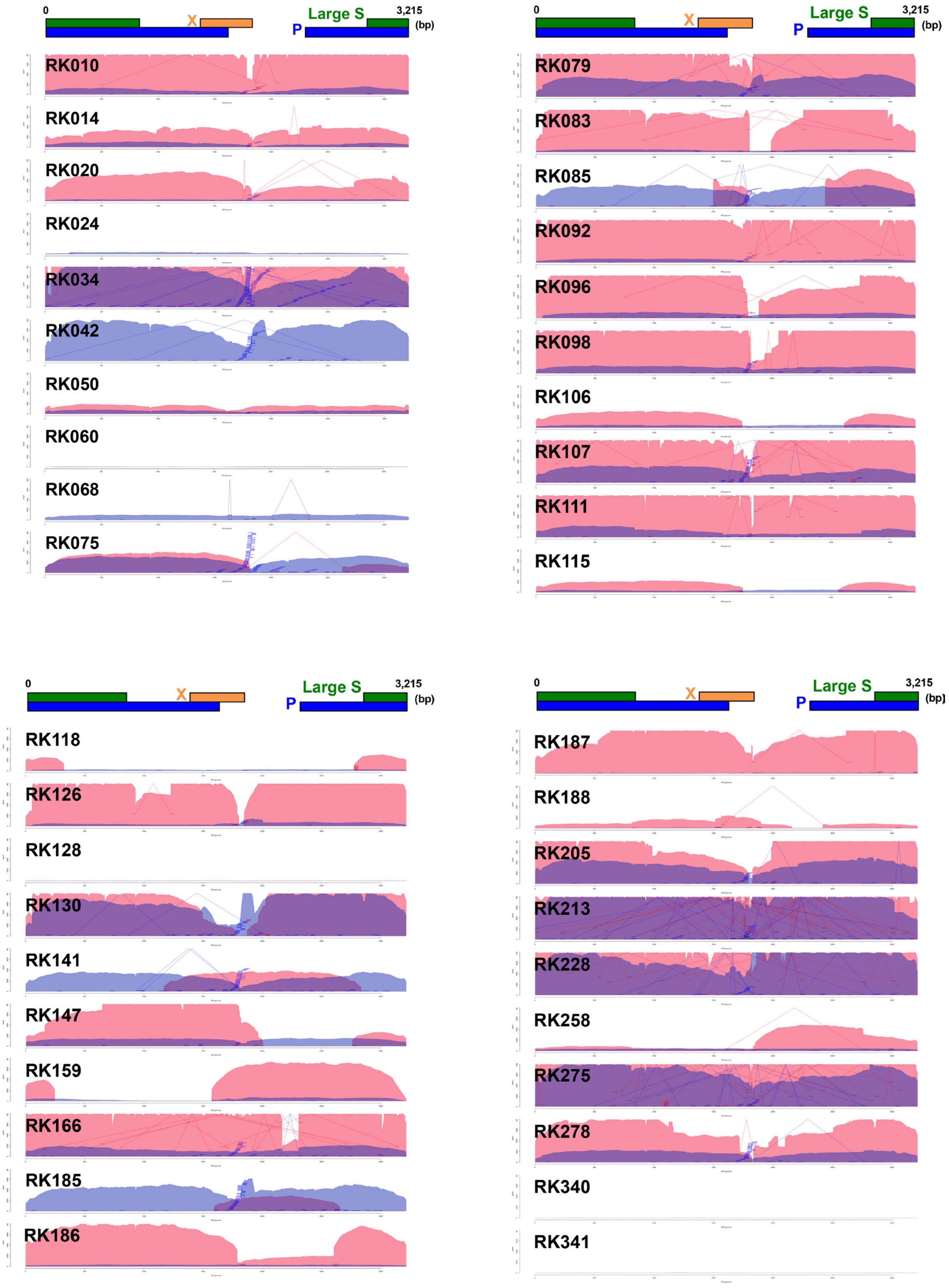

Supplementary Figure 3: Depth of coverage for HBV genome and integration sites in HBV-Capture-Seq. Blue and pink indicate the HBV depth in non-tumor and tumors. Blue and red bars indicate junctions in liver and cancers. Junctions of HBV are shown as vertical bars, with part of the remaining integrated sequence (35bp) indicated as horizontal lines. Positions of structural variations within HBVs were shown as connected lines.

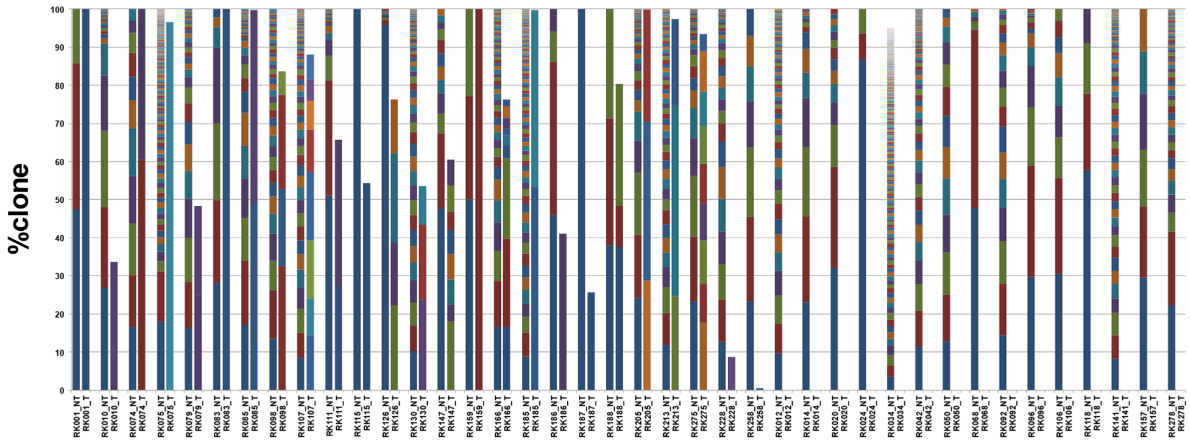

Supplementary Figure 4: Clonal proportion of HBV junctions. Proportion of each junctions detected in NT were shown. Same colored block indicates the shared junction between NT and T.

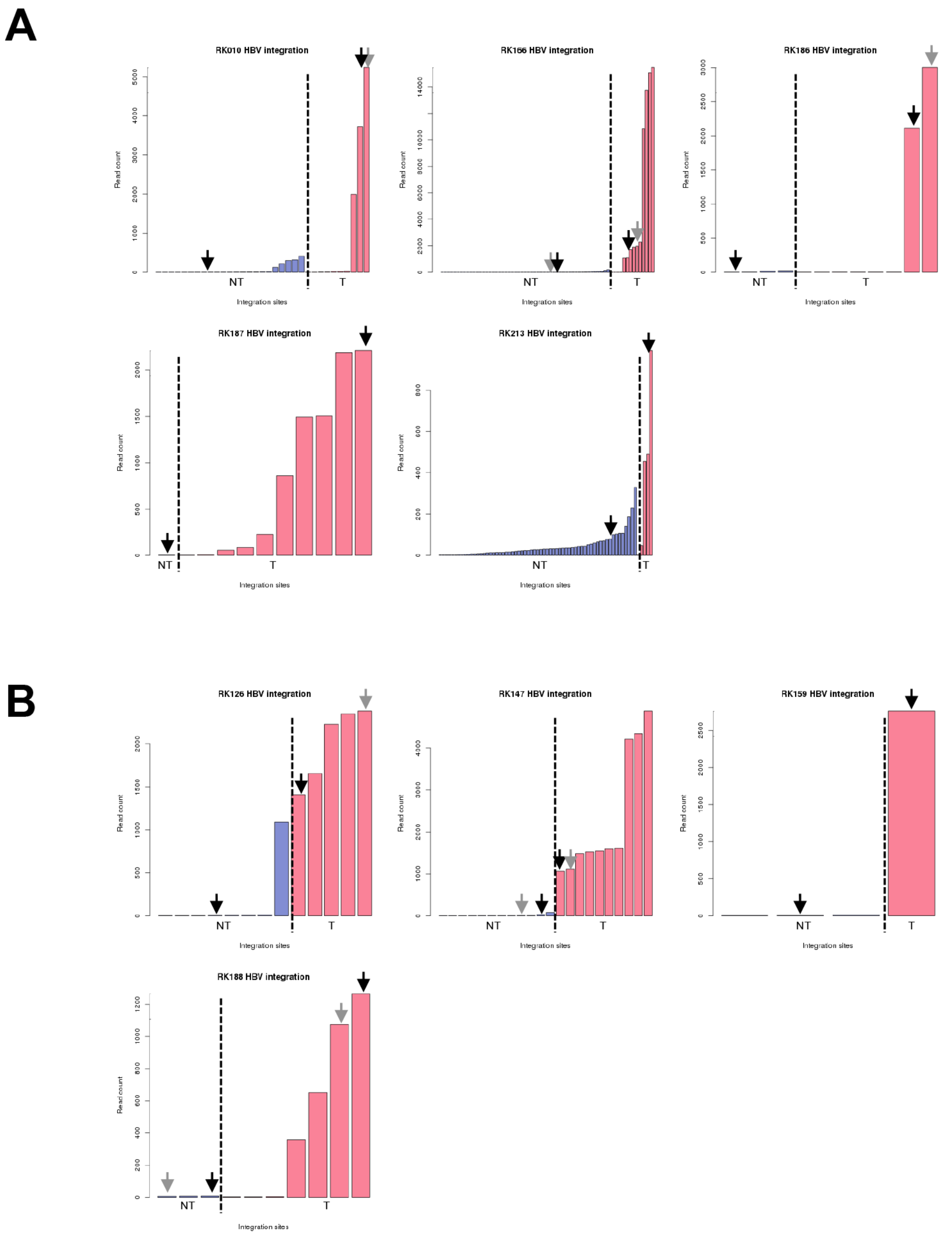

Supplementary Figure 5: Read counts of human-HBV junctions detected in non-tumor (NT, *blue bars*) and tumors (T, *pink bars*) in samples harboring HBV integrations in *TERT* **(A)** and *MLL4* **(B)** in both NT and T. Arrows indicate junctions in *TERT* or *MLL4*. Black and gray arrows are 5' or 3' side pairs of junctions.

Supplementary Table 1: Sample list of HBV infection model of human hepatocyte chimeric mice

Sample ID	DNA sample	Days after infection	Description	HBV copy (IU/mL)
Day 0_#1	PXB0264-0004	None	liver DNA	ND
Day 0_#2	PXB0264-0005	None	liver DNA	ND
Day 10_#1	PXB0282-0025	10	liver DNA	9.00E+05
Day 10_#2	PXB0282-0026	10	liver DNA	2.50E+05
Day 10_#3	PXB0282-0003	10	liver DNA	9.60E+04
Day 10_#4	PXB0282-0003	10	liver DNA	9.60E+04
Day 23_#1	HSB0109-0025_1	23	liver DNA	1.77E+04
Day 23_#2	HSB0109-0025_2	23	liver DNA	1.77E+04
Day 23_#3	HSB0109-0072_1	23	liver DNA	1.00E+03
Day 23_#4	HSB0109-0072_2	23	liver DNA	1.00E+03
Day 23_#5	HSB0109-0074_1	23	liver DNA	1.61E+03
Day 23_#6	HSB0109-0074_2	23	liver DNA	1.61E+03
Day 49_#1	HSB0109-0005_1	49	liver DNA	9.91E+04
Day 49_#2	HSB0109-0005_3	49	liver DNA	9.91E+04
Day 49_#3	HSB0109-0069_1	49	liver DNA	3.16E+04
Day 49_#4	HSB0109-0069_2	49	liver DNA	3.16E+04
Day 49_#5	HSB0109-0070_1	49	liver DNA	4.86E+04
Day 49_#6	HSB0109-0070_2	49	liver DNA	4.86E+04
Day 56_#1	PXB0282-0004	56	liver DNA	1.40E+10
Day 56_#2	PXB0282-0001	56	liver DNA	1.00E+10
Day 56_#3	PXB0282-0005_1	56	liver DNA	6.1E+09
Day 56_#4	PXB0282-0005_2	56	liver DNA	6.1E+09
Day 100_#1	PXB0317-0070	100	liver DNA	5.00E+08
Day 100_#2	PXB0200-063	100	liver DNA	4.80E+07
Day 100_#3	PXB0244-0042	100	liver DNA	1.9E+08
Day 0_HBV10c	PXB0264-0005	None	liver DNA + HBV DNA	ND
Day 0_HBV100c	PXB0264-0005	None	liver DNA + HBV DNA	ND

Supplementary Table 2: HBV integrations in human hepatocyte chimeric mice infected with HBV

See Supplementary File 1

Supplementary Table 3: Clinical and pathological information of humna liver tissues

See Supplementary File 1

Supplementary Table 4: Summary of HBV-capture sequencing

See Supplementary File 1

Supplementary Table 5: HBV integrations in HBV infected samples

See Supplementary File 1

Supplementary Table 6: List of gene-related regions harboring shared junctions between NT and T

Gene name	Number of cases	Case names*
TERT	5	RK010 (p), RK166 (i), RK186 (i), RK187 (i),RK213 (i)
KMT2B(MLL4)	4	RK126 (e), RK147 (e), RK159 (e), RK188(i)
ADAMTS6	1	RK098 (i)
ANKRD17	1	RK075 (i)
C1orf168	1	RK130 (i)
CCNA2	1	RK107 (i)
CPA6	1	RK147 (i)
DOK5	1	RK098 (i)
EML4	1	RK111 (i)
FSIP2	1	RK205 (i)
HEATR6	1	RK079 (e)
HPR	1	RK130 (p)
LOC100506421	1	RK107 (i)
MAP4K2	1	RK213 (p)
NKAIN3	1	RK085 (i)
PLXDC2	1	RK147 (i)
REXO1	1	RK275 (i)
RGS12	1	RK126 (i)
SEMA3C	1	RK228 (i)
SEMA6B	1	RK107(e)
SFMBT2	1	RK083 (i)
THSD4	1	RK107 (i)
TPPP	1	RK115 (i)
USP9Y	1	RK205 (i)

*(p): promoter, (i):intron, (e):exon

Supplementary Table 7: List of integrations in genes with fusion transcripts

Gene name	Numbers of cases with*		Sample names
	integrations	fusions	
FN1	14	8	RK014_NT, RK034_NT, RK079_NT, RK085_NT, RK098_NT, RK107_NT, RK130_NT, RK228_NT
KMT2B(MLL4)	6	5	RK014_T, RK092_T, RK126_T, RK147_T, RK159_T
TERT	9	2	RK010_T, RK187_T
ALB	2	2	RK034_NT, RK092_NT
LOC100506421	3	1	RK107_T
DOK5	2	1	RK098_T
HS6ST3	2	1	RK096_T
C1orf168	2	1	RK130_T
CCNA2	2	1	RK107_T
CPA6	1	1	RK147_T
RGS12	1	1	RK126_T
ATXN7L1	1	1	RK213_NT
RB1CC1	1	1	RK106_T
PPP2R2B	1	1	RK034_NT
VKORC1L1	1	1	RK012_NT
CDK15	1	1	RK050_T
RBP4	1	1	RK130_NT
NOS1	1	1	RK079_NT
USP9Y	1	1	RK205_T
GRIA1	1	1	RK034_NT
PRKG1	1	1	RK075_NT
CDK13	1	1	RK141_NT
TPPP	1	1	RK115_T
NUP153	1	1	RK107_NT
KHDRBS3	1	1	RK141_NT
KCNIP4	1	1	RK141_NT
TEKT3	1	1	RK098_T

*Cases number were caliculated for samples with available RNA-Seq data.
